# Tyrosine Phosphorylation of the K_v_2.1 Channel Contributes to Injury in Brain Ischemia

**DOI:** 10.3390/ijms21249538

**Published:** 2020-12-15

**Authors:** Min-Young Song, Ji Yeon Hwang, Eun Ji Bae, Saesbyeol Kim, Hye-Min Kang, Yong Jun Kim, Chan Park, Kang-Sik Park

**Affiliations:** 1 Department of Physiology, College of Medicine, Kyung Hee University, Seoul 02447, Korea; kn60820@naver.com (M.-Y.S.); wldusdl314@naver.com (J.Y.H.); bag3334@naver.com (E.J.B.); totquf05@naver.com (S.K.); 2 Department of Anatomy & Neurobiology, College of Medicine, Kyung Hee University, Seoul 02447, Korea; mkang@khu.ac.kr (H.-M.K.); psychan@khu.ac.kr (C.P.); 3 Department of Pathology, College of Medicine, Kyung Hee University, Seoul 02447, Korea; yjkim1@khu.ac.kr; 4 KHU-KIST Department of Converging Science and Technology, Kyung Hee University, Seoul 02447, Korea

**Keywords:** K_v_2.1, tyrosine phosphorylation, brain ischemia, oxidative stress

## Abstract

In brain ischemia, oxidative stress induces neuronal apoptosis, which is mediated by increased activity of the voltage-gated K^+^ channel K_v_2.1 and results in an efflux of intracellular K^+^. The molecular mechanisms underlying the regulation of K_v_2.1 and its activity during brain ischemia are not yet fully understood. Here this study provides evidence that oxidant-induced apoptosis resulting from brain ischemia promotes rapid tyrosine phosphorylation of K_v_2.1. When the tyrosine phosphorylation sites Y124, Y686, and Y810 on the K_v_2.1 channel are mutated to non-phosphorylatable residues, PARP-1 cleavage levels decrease, indicating suppression of neuronal cell death. The tyrosine residue Y810 on K_v_2.1 was a major phosphorylation site. In fact, cells mutated Y810 were more viable in our study than were wild-type cells, suggesting an important role for this site during ischemic neuronal injury. In an animal model, tyrosine phosphorylation of K_v_2.1 increased after ischemic brain injury, with an observable sustained increase for at least 2 h after reperfusion. These results demonstrate that tyrosine phosphorylation of the K_v_2.1 channel in the brain may play a critical role in regulating neuronal ischemia and is therefore a potential therapeutic target in patients with brain ischemia.

## 1. Introduction

Oxidative stress has been shown to be a major risk factor for neuronal apoptosis and neurodegenerative diseases, as seen with Alzheimer’s and brain ischemia [1,2,3]. Neuronal cell death is mainly attributable to apoptosis, although cell death mechanisms are complicated and diverse [4]. The oxidative stress seen in ischemia in mammalian neurons is accompanied by enhancement of the K^+^ current [5]. This enhancement results in an efflux of K^+^ and is an essential factor for neuronal apoptosis [6]. Additionally, a number of studies have reported that caspase activation, the mitochondrial membrane potential, and overall cellular volume are regulated by the necessary abundance of intracellular K^+^ levels, in addition to cell osmolality [7]. It is understandable then that K^+^ efflux initiates the apoptotic cascade, indicated by cell shrinkage and mitochondrial cytochrome-c release [8]. Further K^+^ loss after any apoptotic stimulus can be caused by increased activation of voltage-gated K^+^ (K_v_) channels [6]. Blocking K_v_ channels effectively attenuates cell death in many apoptotic models, which can be pharmacologically accomplished through the administration of staurosporine, an apoptotic drug [8]. This suggests that suppression of the K^+^ efflux via K_v_ channels can inhibit the apoptosis stimulated by oxidative stress [9].

K_v_2.1 is a major component of the delayed rectifier K^+^ channel in the pyramidal neurons of the cortex and hippocampus. It is highly phosphorylated in the brain and is expressed in large clusters, which are restricted to soma proximal dendrites and the axon initial segment [10,11]. The calcineurin-mediated dephosphorylation of K_v_2.1 leads to a hyperpolarizing shift of voltage-dependent biophysical properties and to the dispersion of clusters, both of which may suppress the neuronal cell death caused by excitotoxic injury by decreasing neuronal excitability [12,13,14].

Y124, the N-terminal tyrosine residue of K_v_2.1, has previously been shown to be critical in the apoptotic current surge [15]. This site is rapidly increased by oxidant stimulation and is phosphorylated by Src kinase. We recently used mass spectrometry to demonstrate that the K_v_2.1 channel contains two tyrosine phosphorylation sites (Y686 and Y810) within the C-terminal intracellular region, that these sites are regulated by Src kinase, and that they are physiologically important for channel activity [16].

Recently, it has been reported that K_v_2.1 channel may be a target of therapy in ischemic brain injuries [17], therefore further investigations are needed for its precise role. The specific acting mechanisms regulating K_v_2.1 tyrosine phosphorylation under pathological conditions remain unclear, although it has been shown that alteration of K_v_2.1 tyrosine phosphorylation has important ramifications for neuronal apoptosis [18]. In the present study, we showed that brain ischemia increases tyrosine phosphorylation of the K_v_2.1 channel, inducing neuronal apoptosis, and that the Y686 and Y810 residues in the K_v_2.1 channel play critical roles in oxidative stress-induced ischemia. Taken together, our results suggest that tyrosine phosphorylation of K_v_2.1 is critical for regulating brain ischemia.

## 2. Results

### 2.1. Oxidative Stress Induces Tyrosine Phosphorylation of K_v_2.1

**Figure 1:**
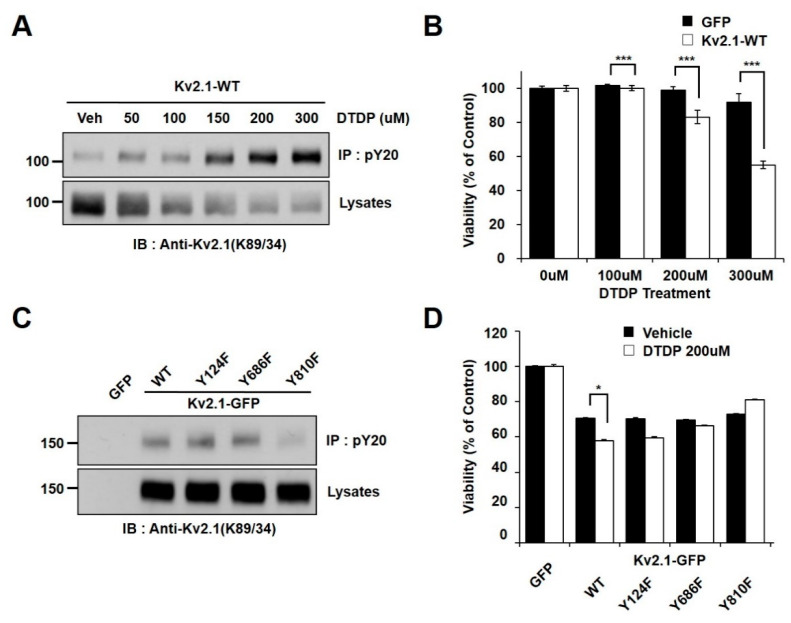
Tyrosine phosphorylation of the K_v_2.1 channel is induced by DTDP treatment. (**A**) The concentration-dependent effect of DTDP on K_v_2.1 tyrosine phosphorylation. HEK293 cells were transfected with the K_v_2.1 plasmid. Immunoblot showing that tyrosine phosphorylation levels of the K_v_2.1 channel protein are induced in a concentration-dependent manner (0–300 µM) by DTDP treatment. (**B**) Cell viability assay demonstrating the effect of DTDP in HEK293 cells expressing either GFP (black bar) or K_v_2.1 (white bar). (**C**) HEK293 cells were transfected with K_v_2.1-WT or tyrosine mutants (Y124F, Y686F, or Y810F). No difference was detected between the WT and mutants for the expression of the K_v_2.1 channel after DTDP treatment (200 µM). The Y810 mutation decreased tyrosine phosphorylation of K_v_2.1. (**D**) Cell viability assay showing the mean effect of DTDP (200 µM) on viability in HEK293 cells expressing the K_v_2.1-WT or K_v_2.1 mutations. Data are expressed as the mean ± S.E.M. (* *p* < 0.05, *** *p* < 0.01 in Student’s *t* test).

In an effort to determine whether oxidative stress is associated with K_v_2.1 channel tyrosine phosphorylation, we observed the phosphorylation changes in HEK293 cells transiently expressing the rat K_v_2.1 channel. The cells were treated with increasing concentrations of 2,2′-dithiodipyridine (DTDP), a sulfhydryl oxidizing agent. The channel proteins were immunopurified with an anti-phosphotyrosine (PY20) and analyzed by immunoblotting with an anti-K_v_2.1 (K89/34) mAb in order to detect the tyrosine phosphorylated K_v_2.1 channel. We found that the administration of DTDP treatment in a concentration-dependent manner decreased the expression levels of the K_v_2.1 channel protein, whereas the tyrosine phosphorylation levels of the K_v_2.1 channel were shown to be significantly increased (Figure 1A). We next examined the viability of cells under oxidative stress due to the presence or absence of the K_v_2.1 channel. The vulnerability of these cells to DTDP- induced death significantly increased in the K_v_2.1-expressing cells (Figure 1B).

We used mass spectrometry in previous studies to identify two novel tyrosine phosphorylation sites (Y686 and Y810) on the K_v_2.1 channel, also showing that the Y124, Y686, and Y810 residues on K_v_2.1 are directly phosphorylated by Src kinase and are involved in the K_v_2.1 channel activity [16]. Moreover, Src kinase activity has been proven to rise after DTDP-induced cell death, while tyrosine phosphorylation of the K_v_2.1 channel is induced after oxidative stress through activated Src kinases [19]. Here, we performed a comparative analysis of the role of the WT K_v_2.1 channel with that of the K_v_2.1 channel with non-phosphorylatable mutations in response to DTDP-induced cell death. We transfected pEGFP-C1, K_v_2.1-WT-GFP, and three non-phosphorylatable mutants (K_v_2.1-Y124F-GFP, K_v_2.1-Y686F-GFP, and K_v_2.1-Y810F-GFP) into HEK293 cells. Cells were then treated with DTDP (200 µM, 10 min). The tyrosine phosphorylated K_v_2.1 protein was then immunopurified with an anti-PY20 mAb, before undergoing immunoblotting with an anti-K_v_2.1 (K89/34) mAb. The DTDP-induced tyrosine phosphorylation levels were not different between the K_v_2.1-WT, K_v_2.1-Y124F, and K_v_2.1-Y686F channels, whereas the tyrosine phosphorylation levels of the K_v_2.1-Y810F mutation decreased significantly compared to levels seen in the K_v_2.1-WT and other channel mutations. However, despite replacing each tyrosine residues (Y124, Y686, or Y810) with phenylalanine, the overall expression levels of the K_v_2.1 mutations were not different from that of the K_v_2.1-WT (Figure 1C). Cells were treated with the oxidant DTDP (200 µM, 10 min) and then subjected to cell viability analyses with WST-1 reagent. The cell viability analysis of the DTDP-treated cells showed that viability decreased in cells expressing K_v_2.1-WT, K_v_2.1-Y124F, and K_v_2.1-Y686F compared to GFP-expressing cells, whereas viability was increased in cells expressing the K_v_2.1-Y810F mutation (Figure 1D). The results of the cell viability and immunochemical assays were similar for viability and K_v_2.1 tyrosine phosphorylation levels in DTDP-treated cells. These results suggest that increased tyrosine phosphorylation levels of K_v_2.1 may be a major contributor to DTDP-induced cell death, and the Y810 residue is a key regulatory phosphorylation site.

### 2.2. Regulation of Apoptotic Cell Death by Tyrosine Phosphorylation of K_v_2.1

**Figure 2:**
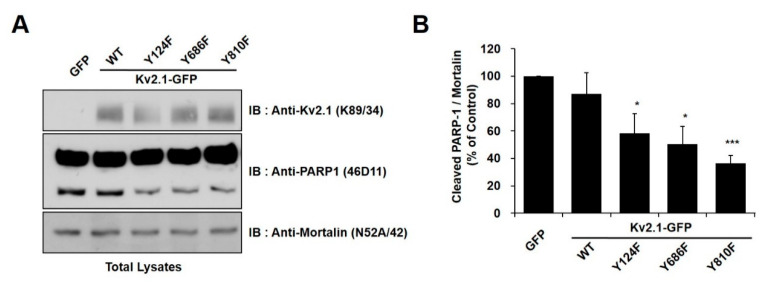
Tyrosine mutations of the K_v_2.1 channel decreased apoptotic cell death in DTDP-treated cells. (**A**) Immunoblot analysis of PARP-1 cleavage in HEK293 cells expressing K_v_2.1-WT or the mutations after DTDP treatment. The full-length PARP-1 (116 kDa) and the fragmented PARP-1 (89 kDa) apoptotic cleaved product as well as mortalin, which was used as a loading control. (**B**) Compared with K_v_2.1-WT, DTDP treatment decreased cleaved PARP-1 expression in cells containing the K_v_2.1 channel tyrosine mutations. Data are expressed as the mean ± S.E.M. (* *p* < 0.05, *** *p* < 0.01, Student’s *t* test).

In order to identify the tyrosine phosphorylation sites (Y124, Y686, or Y810) on the K_v_2.1 channel that are associated with DTDP-induced apoptotic cell death, we treated cells expressing the mutations K_v_2.1-Y124F, K_v_2.1-Y686F, and K_v_2.1-Y810F with the oxidizing agent DTDP (200 µM, 10 min). We observed the expression of cleaved PARP-1 protein as an apoptotic marker. All of the DTDP-treated cells showed a striking decrease in the expression of the cleaved PARP-1 protein (Figure 2A). Similar to the results of the viability tests, the tyrosine mutants of K_v_2.1-expressing cells showed a decrease in cleaved PARP-1 protein, when compared with the K_v_2.1-WT-expressing cells. Particularly, K_v_2.1-Y810F-expressing cells showed approximately a 35% decrease in cleaved PARP-1 protein (Figure 2B). These results indicate that the Y810 residues of the K_v_2.1 channel may play an important role in apoptotic cell death caused by oxidative stress.

### 2.3. Brain Ischemia Results in Tyrosine Phosphorylation of the K_v_2.1 Channel

**Figure 3:**
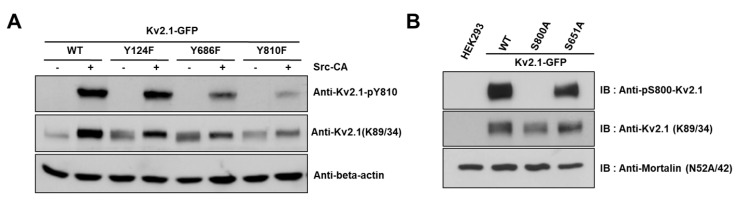
Analysis of the K_v_2.1 channel tyrosine phosphorylation sites. Phospho-specific tyrosine antibodies were generated against the S800 phosphorylation site (anti-pS800-K_v_2.1, in-house) and the Y810 phosphorylation site (anti-pY810-K_v_2.1, in-house) on the K_v_2.1 channel. (**A**) HEK293 cells were co-transfected with K_v_2.1-WT or the channel mutations (Y124F, Y686F, and Y810F) and Src-CA. Immunoblot analyses were performed against extracts of HEK293 cells expressing the K_v_2.1-WT and the tyrosine phosphorylation site mutations. Extracts were normalized by comparing immunoreactivity of the total K_v_2.1 with that for the general anti-K_v_2.1 (K89/34). (**B**) Immunoblot analyses performed against extracts from HEK293 cells expressing K_v_2.1-WT, and the respective S800A and S651A mutants with anti-pS800 K_v_2.1 and the general anti-K_v_2.1 (K89/34).

We generated an anti-pY810-K_v_2.1 to detect the phospho-Y810 residue of K_v_2.1, thus elucidating whether the previously identified tyrosine phosphorylation sites on the HEK293 K_v_2.1 channel [16] are phosphorylated on the native K_v_2.1 channel expressed in the ischemic brain (Figure 3A).

**Figure 4:**
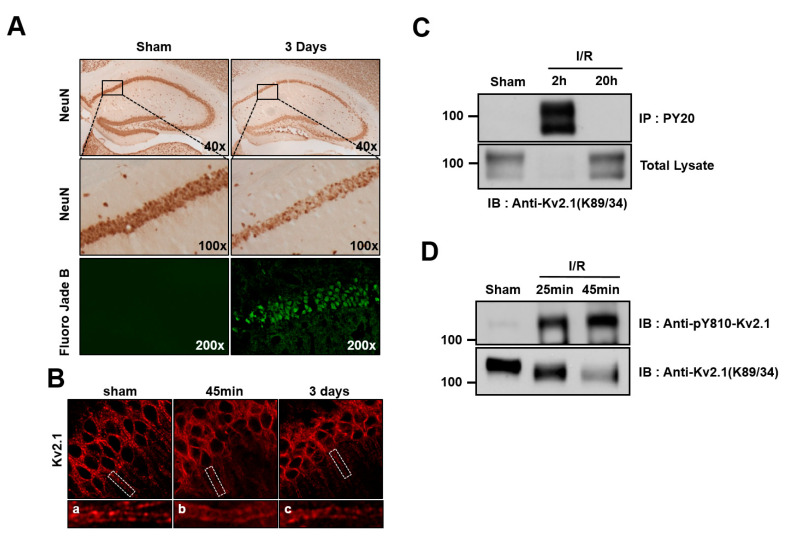
Tyrosine phosphorylation of the K_v_2.1 channel after ischemia/reperfusion. (**A**) Representative photographs of brain sections labeled with NeuN and Fluoro-Jade B (green) 3 days after ischemia/reperfusion injury (20 min). NeuN labeling (top and middle panel) of the hippocampus in control rats and those with ischemia/reperfusion injuries. Middle panel is the magnification of the boxed areas shown in the top panel. Cells positively stained for Fluoro-Jade B were identified as apoptotic neurons. Fluoro-Jade B-labeled cells (bottom panel) were present in CA1 pyramidal neurons undergoing apoptosis. (**B**) Clustering of the K_v_2.1 channel on the soma and dendrites of a control rat brain (left). Dispersion of K_v_2.1 clusters 45 min after ischemia/reperfusion (middle). Three days after ischemia/reperfusion, the clustering of K_v_2.1 was restored (right). Panels a-c are magnified views of the boxed areas in the top panel. (**C**) Brain membrane fractions isolated from control rats and rats subjected to 4 VO 2 and 20 h after ischemia/reperfusion were immunoprecipitated with anti-pY20. The immunoprecipitates were separated by 7.5% SDS-PAGE and immunoblotted with anti-K_v_2.1 mAb. (**D**) Membrane lysates from the hippocampus isolated from control mice and mice subjected to two-vessel occlusion 25 min and 45 min after ischemia/reperfusion were immunoblotted with anti-pY810-K_v_2.1 or anti-K_v_2.1-mAb.

To that end, we analyzed the brain sections of two-vessel occlusion mice killed 45 min or 3 days after 20 min of global ischemia. After 3 days of reperfusion, brains were perfusion-fixed with paraformaldehyde, and NeuN or Fluoro-Jade B staining was performed in order to discover the surviving neurons and apoptotic cells in the hippocampus CA1 region. In the ischemia group, the number of NeuN-positive neurons was shown to have decreased compared with the control, whereas Fluoro-Jade B-positive apoptotic cells had significantly increased (Figure 4A). Previous studies demonstrated that K_v_2.1 channels form distinct clusters that are restricted to the neuronal cell membrane of the somatodendritic regions. In addition, pathogenic conditions, such as brain ischemia and spinal cord injury, lead to K_v_2.1 dispersion [12,20]. Thus, we went on to determine whether K_v_2.1 tended to cluster in the hippocampal CA1 region. We found that the K_v_2.1 channel was entirely dispersed over the somatodendritic membrane of neurons 45 min after brain ischemia. However, clustering of the K_v_2.1 channel was observed three days after ischemia (Figure 4B).

In order to determine whether ischemic brain injury mediates tyrosine phosphorylation on the K_v_2.1 channel, we performed immunoblotting and immunoprecipitation analyses on the ischemia-injured brain tissue of rats that had undergone four-vessel occlusion using tyrosine phospho-specific K_v_2.1 channel antibodies. After 20 min of ischemia and the subsequent reperfusion, the K_v_2.1 channel protein was immunopurified with PY20 mAb from the brain tissue of the control animals, as well as from brains 2 and 20 h after undergoing experimentally induced ischemia. The total protein levels of the K_v_2.1 channel decreased 2 h after ischemia but were already restored to levels seen in the control by 20 h after ischemia. Importantly, although K_v_2.1 protein expression had significantly decreased 2 h after ischemia, the tyrosine phosphorylation level of the K_v_2.1 channel was markedly increased at the same time, whereas the tyrosine phosphorylation of the channel could not be detected 20 h after brain ischemia (Figure 4C). We then investigated whether the Y810 residue of K_v_2.1 was phosphorylated as a result of acute ischemic brain injury in two-vessel occlusion mice. After reperfusion, the total K_v_2.1 protein level decreased, in rat brain membranes whereas the tyrosine phosphorylation level markedly increased (Figure 4D). These results were consistent with our immunostained brain tissue findings, demonstrating that acute ischemic stress enhanced K_v_2.1 tyrosine phosphorylation, irrespective of the methods used.

### 2.4. Y810 Phosphorylation Affects p38-Mediated Phosphorylation of K_v_2.1 at S800

**Figure 5:**
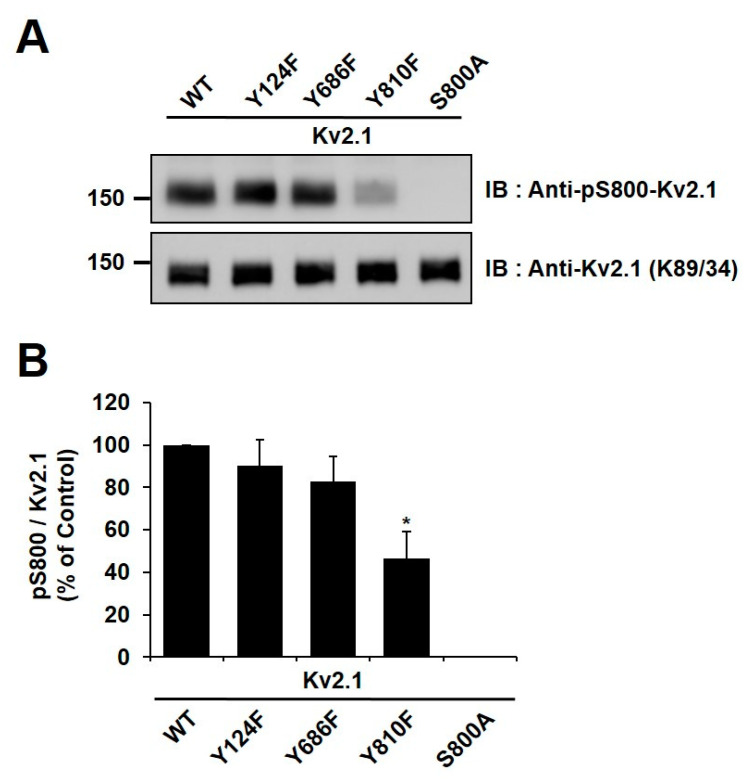
Phosphorylation sites Y810 and S800 of the K_v_2.1 channel were mutually co-regulated during oxidative stress. (**A**) HEK293 cells were transfected with K_v_2.1-WT or the K_v_2.1 channel tyrosine mutations (Y124F, Y686F, and Y810F). The cells were treated with DTDP (200 µM, 10 min), lysed, and immunoblotted with anti-K_v_2.1 mAb and anti-pS800-K_v_2.1 pAb. (**B**) Quantification of phosphorylated S800 (pS800) in DTDP-treated cells containing the Y124F, Y686F, and Y810F channel mutations. The phosphorylation level of S800 (pS800) in the cells expressing channel mutations is presented as a ratio of pS800 to total K_v_2.1 protein and normalized to K_v_2.1-WT control. Data are expressed as the mean ± S.E.M. (* *p* < 0.05, Student’s *t* test).

It has been reported in previous studies that the S800 residue of the K_v_2.1 channel is directly phosphorylated by p38 MAPK during apoptosis and that Src directly influences the phosphorylation of this residue [9,18]. In order to examine whether Y810 phosphorylation influences the phosphorylation of S800, we generated an anti-pS800 K_v_2.1 (Figure 3B) and then expressed K_v_2.1-Y124F, K_v_2.1-Y686, and K_v_2.1-Y810 mutations in HEK293 cells. We then analyzed the mutants by immunoblotting with anti-K_v_2.1 (K89/34) mAb and anti-pS800-K_v_2.1. The S800 v2.1 residue was shown to be clearly phosphorylated following oxidant treatment in the K_v_2.1-WT, whereas S800 was only weakly phosphorylated in cells expressing the K_v_2.1-Y810F mutation (Figure 5A,B). These findings indicate that the Y810 phosphorylation site on the K_v_2.1 channel is a key regulatory site and may affect the function of the S800 residue in oxidative stress-induced apoptosis.

## 3. Discussion

K_v_2.1 channel dephosphorylation and cluster dispersion as a result of altered neuronal activity are elicited by excitatory stimuli like ischemia, spinal cord injury, seizures in vivo, or by glutamate treatment, serum deprivation, or oxidative stress, in vitro [5,20,21,22]. A role for K_v_2.1 has been reported as a pro-apoptotic neuronal trigger, but the mechanism for this is not fully understood. In the present work, we explored the role of K_v_2.1 tyrosine phosphorylation in the neuronal apoptosis induced by brain ischemia.

We found that the K_v_2.1 channel undergoes rapid tyrosine phosphorylation after oxidative stress in transfected HEK293 cells and in neurons after global brain ischemia (Figure 1A and Figure 4C,D). It has previously been shown that ischemia regulates the rapid tyrosine phosphorylation of the K_v_1.2 channel and that persistent neuronal depolarization and enhanced intracellular calcium and zinc concentrations are induced by ischemia [23]. Convergent calcium and zinc signaling regulate this apoptotic K_v_2.1 channel, and its tyrosine phosphorylation and activity are increased with increasing intracellular zinc concentrations [18,22,24]. Moreover, K_v_2.1 is functionally modulated by zinc and calcium in response to ischemia [13,25]. Therefore, the elevation of intracellular calcium and zinc concentrations induced by ischemic injury in neurons after brain ischemia [26] may lead to tyrosine phosphorylation of K_v_2.1 within a short time (Figure 4C,D). We also found that tyrosine phosphorylation of K_v_2.1 was no longer present 20 h after ischemia (Figure 4C). In other words, neuronal K_v_2.1 was transiently tyrosine phosphorylated after brain ischemia. This observation is supported by previous studies reporting that intracellular calcium levels and the activity of the Src family kinases (SFKs) are increased by brain ischemia [27]. The SFK pathway is also involved in neuronal cell apoptosis in response to oxidative stress conditions [22,28]. We had previously observed that the K_v_2.1 channel is tyrosine phosphorylated and that the K^+^ efflux is induced by Src kinase [16]. Therefore, it is reasonable to assume that tyrosine phosphorylation of the K_v_2.1 channel after brain ischemia is regulated by Src kinase.

Several studies have shown that mutating specific tyrosine phosphorylation residues bestows in neuroprotective and anti-apoptotic effects [29,30]. Src kinase activity is induced by oxidative stress [31] and we previously reported that the Y124, Y686, and Y810 residues of the K_v_2.1 channel are directly phosphorylated by Src kinase [16]. In the present work, we found that a mutation of Y810F in the K_v_2.1 channel showed a significant decrease in tyrosine phosphorylation during oxidative stress (Figure 1C). Moreover, the cells expressing K_v_2.1-Y810F showed a higher survival rate during oxidative stress than cells with other mutations (Figure 1D). Thus, we can conclude that Y810 phosphorylation on K_v_2.1 may be a major contributor to oxidative stress-induced apoptosis.

We also showed that K_v_2.1 clustering was dispersed after brain ischemia (Figure 4B). It has already been shown that the clustering of the K_v_2.1 channel is indicative of a restricted localization in the somatodendritic plasma membrane [21], although the specific role of K_v_2.1 declustering and its association with neuronal apoptosis in brain ischemia is not fully understood. The K_v_2.1 channel is also localized to lipid drafts in the brain, and the current density and location of the K_v_2.1 channels in lipid rafts are altered by cholesterol depletion of the cell membrane [32]. We previously reported that K_v_2.1 channel activity is dynamically changed by Src-mediated tyrosine phosphorylation [16]. Thus, it will be important to conduct future studies that can help clarify whether tyrosine phosphorylation of K_v_2.1 can regulate the channel clustering in the plasma membrane.

The present work showed that the Y124, Y686, and Y810 residues of the K_v_2.1 channel are involved after they are phosphorylated during oxidative stress-induced neuronal apoptosis. The coordination of Y124 and S800 residues has been reported to regulate the channel activity of K_v_2.1 in oxidative stress-induced apoptosis [22]. The Y124 and S800 residues of K_v_2.1 are phosphorylated by Src and p38 kinase, respectively. Additionally, p38 kinase is activated via apoptosis signal-regulating kinase 1 in oxidant-stimulated zinc release [22,33]. Oxidative stress-induced phosphorylation of S800 increases K_v_2.1 currents, blocking toxicity through p38 kinase inhibition [9]. Src kinase-mediated Y124 phosphorylation is inhibited by the cytoplasmic protein tyrosine phosphatase ε (Cyt-PTPε) [15]. Inhibition of Src kinase activity blocks the apoptotic K^+^ current surge and overexpression of Cyt-PTPε inhibits K^+^ current, thus performing a neuroprotective function [15,22]. Src and p38 kinase-mediated K_v_2.1 phosphorylation have been suggested as regulators of K^+^ current and cell survival during apoptosis [18]. In the present study, in agreement with previous results [18,22], Y124, Y686, and Y810 K_v_2.1 mutations decreased the phosphorylation of S800 under the pathological conditions induced by oxidant treatment, with the Y810 mutation serving as the predominant blocker of S800 phosphorylation (Figure 5). Therefore, coordinating the phosphorylation of Y810 may regulate the activity of the K_v_2.1 channel after ischemia, and thus neuronal apoptosis. Our findings also suggest that the K_v_2.1 channel Y810 residue can be added to the list of ischemic regulatory factors mediated by Src kinase.

In summary, we demonstrated that oxidative stress-induced neuronal apoptosis promoted the K_v_2.1-Src kinase signaling pathway, decreased the expression of the K_v_2.1 channel protein, and increased the tyrosine phosphorylation of K_v_2.1. Most importantly, the Y810 residue of the K_v_2.1 channel was found to be a critical site for phosphorylation in oxidative stress-induced neuronal apoptosis. Our findings support the potential pharmacological targeting of the K_v_2.1 channel, which will likely be beneficial in combatting brain damage. Therefore, inhibiting the K_v_2.1 tyrosine phosphorylation induced by ischemia may be a therapeutic target during early neuronal apoptotic conditions.

## 4. Materials and Methods

### 4.1. Cell Culture and Transient Transfection

The HEK293 cells were grown in Dulbecco’s modified Eagle’s medium (DMEM, Welgene, Gyeongsan-si, Korea), supplemented with 10% fetal bovine serum (Welgene, Gyeongsan-si, Korea), and a 1% penicillin-streptomycin solution (Welgene, Gyeongsan-si, Korea). Cells were cultured at 37 °C in a humidified atmosphere containing 95% air and 5% CO2 and passaged when 80% confluent. Transient transfections were performed with either the K_v_2.1 wild-type (WT) channel or Y124F, Y686F, and Y810F mutations using pEGFP-N1 plasmids with Lipofectamine 2000 reagent (Invitrogen, Grand Island, NY, USA).

### 4.2. Immunoblotting

Cultured HEK293 cells were washed with ice-cold phosphate-buffered saline (PBS), before being lysed in buffer containing 1% Triton X-100, 150 mM NaCl, 50 mM Tris-HCl (pH 8.0), 2 mM sodium orthovanadate, 5 mM NaF, 5 mM sodium pyrophosphate, aprotinin (1.5 μg/mL), antipain (10 μg/mL), leupeptin (10 μg/mL), and benzamidine (0.1 mg/mL). Lysates were then cleared by centrifugation at 16,100× *g* for 30 min at 4 °C, then were separated by 7.5% SDS-PAGE, transferred to nitrocellulose membranes, and immunoblotted with anti-K_v_2.1 (K89/34, NeuroMab, CA, USA), anti-phosphotyrosine (PY20, Upstate Biotechnology, MA, USA), and in-house phosphospecific antibodies (anti-pan-pY-K_v_2.1, pY810-K_v_2.1, and anti-pS800). The membranes were incubated with goat anti-mouse (Enzo, Ann Arbor, MI, USA) or goat anti-rabbit (Pierce, Rockford, IL, USA) IgG horseradish peroxidase-conjugated secondary antibody in 4% nonfat milk/Tris-buffered saline. The protein bands belonging to the K_v_ channels were detected using an enhanced chemiluminescence reagent (Pierce, Rockford, IL, USA) and the density of the bands was then measured with ImageJ software (National Institutes of Health, Bethesda, MD, USA).

### 4.3. Animals

Adult 8-week-old male C57BL/6 mice or SD rats (Daehan Biolink, Chungbuk, Korea) were used for the animal experiments. All animals were housed in groups in temperature-controlled (20 ± 2 °C) housing with free access to food and water and exposed to a 12-h light/dark cycle. The surgical interventions and postoperative animal care were performed in accordance with the Guidelines and Policies for Rodent Survival Surgery from the Animal Care Committee of Kyung Hee University (Approval Number KHSASP-19-087).

### 4.4. Phospho-Specific Antibodies

Synthetic phosphopeptides containing phosphoserine amino acids (S800, with amino acids 773-808, ESSPLPT(pS)PKFLRPNC and Y810, with amino acids 805–818, RPNCV(pY)SSEGLTGK) or non-phosphopeptides containing serine at the corresponding positions were synthesized by Quality Controlled Biochemicals (AbClon, Seoul, Korea). Phosphopeptides were conjugated to keyhole limpet hemocyanin (1 milligram peptide per milligram of carrier protein) and injected into rabbits for the production of antisera (AbClon, Seoul, Korea). In order to achieve affinity purification, both the phosphopeptides and the non-phosphopeptides were conjugated to SulfoLink coupling gel (Pierce) via their terminal cysteine residues, and phospho-specific antibodies were affinity-purified with a two-step modification of the standard procedures [12]. Briefly, polyclonal sera were passed over the respective phosphopeptide beads and bound and eluted antibodies were immunoadsorbed against the respective non-phosphopeptide beads, in order to remove non-phospho-specific antibodies. Next, phospho-specificity was verified by immunoblot analyses against extracts from HEK293 cells expressing the WT K_v_2.1, as well as the respective phosphorylation site mutants, after verifying the comparable immunoreactivity of the immunoblot samples using a general anti-K_v_2.1 monoclonal antibody (K89/34, Neuromab, CA, USA).

### 4.5. Immunoprecipitation

A crude brain membrane fraction was prepared, as previously described [21]. Animals were killed by rapid decapitation, whereupon the brains were collected and homogenized in buffer (5 mM sodium phosphate, pH 7.4, 320 mM sucrose, 100 mM NaF, and a protease inhibitor cocktail containing 2 μg/mL aprotinin, 2 μg/mL antipain, 1 μg/mL leupeptin, and 10 μg/mL benzamidine). Homogenates were then centrifuged at 800× *g* for 10 min at 4 °C. The supernatants were centrifuged at 38,000× *g* for 90 min at 4 °C. Total membrane protein (4 mg) was solubilized in a lysis buffer (1% Triton X-100, 150 mM NaCl, 1 mM EDTA, 50 mM Tris-HCl, pH 7.4), 1 mM activated sodium orthovanadate, 5 mM NaF, and the protease inhibitor cocktail for 2 h at 4 °C. This was followed by centrifugation at 13,200 rpm for 40 min at 4 °C and the supernatants were incubated with PY20 antibody overnight at 4 °C. Next, 50 μL of protein G-Sepharose beads were added for 2 h at 4 °C. The beads were washed three times in lysis buffer, and the immunopurified proteins were eluted by boiling in an SDS sample buffer.

### 4.6. Two-Vessel Occlusion

A midline incision was made between the neck and sternum, in order to expose the trachea and the right and left common carotid arteries were carefully separated. Cerebral ischemia was induced by clamping both arteries with two miniature artery clips for 20 min. The clips were then removed to allow reperfusion of blood through the carotid arteries. The control mice underwent the same surgical procedure without the artery occlusion. During the surgery, body temperature was monitored with a rectal probe and was maintained at 37.0 °C, using a temperature-controlled Homeothermic Blanket System (Harvard Apparatus, Holliston, MA, USA).

### 4.7. Four-Vessel Occlusion

Both vertebral arteries were heat-cauterized at each alar foramen using a soldering iron (Change-A-tip™ cautery, Bovie Medical Corporation, Purchase, NY, USA) and both common carotid arteries were occluded with vascular clamps for 20 min the following day. During surgery, body temperature was monitored with a rectal probe and was maintained at 37 °C using a temperature-controlled Homeothermic Blanket System (Harvard Apparatus, Holliston, MA, USA).

### 4.8. Immunohistochemistry

In order to analyze the time course of the neurodegeneration in the hippocampal CA1 layer, the animals were allowed to survive for 45 min or 3 days after global ischemia. They were then anesthetized with Zoletil 50 (Virbac, Carros, France) and transcardially perfused with 4% paraformaldehyde in PBS. The fixed brain was subsequently equilibrated in 30% sucrose solution in order to ensure cryoprotection and was sectioned into 30-μm-thick coronal sections using a cryostat (CM1850, Leica, Germany). The tissue sections were washed in PBS and treated for 30 min in a 1% H2O2 PBS solution. The sections were then incubated with anti-NeuN (1:1000, Millipore) to prepare for the analysis of neuronal cell death. They were washed before biotinylated goat anti-mouse IgG (1:200, Vector Laboratories) was used as the secondary antibody. Signals were amplified using an avidin-peroxidase complex (ABC) kit (1:100, Vector Laboratories). After being allowed to react with 3,3′-diaminobenzidine (Sigma-Aldrich Korea), the sections were dehydrated using ethanol and xylene. They were then mounted with neutral resin. For the analysis of neuronal apoptosis, the sections were incubated for Fluoro-Jade B (Histo-Chem, Inc., AR, USA), following the manufacturer’s instructions [34]. In order to analyze K_v_2.1phosphorylation and clustering, the sections were incubated with anti-K_v_2.1 (K89/34, NeuroMab, CA, USA) and in-house phospho-specific antibodies (anti-pan-pY-K_v_2.1). These sections were washed and were probed with Alexa 594-conjugated goat anti-mouse and Alexa 488-conjugated goat anti-rabbit (1:500, Invitrogen) antibodies for 1 h at room temperature. After being washed again, the sections were mounted onto glass slides and cover slipped for microscopic analysis. All images were acquired with the Zeiss LSM 710 confocal microscope (Carl Zeiss, Oberkochen, Germany).

### 4.9. Cell Viability

Transfected cells were grown to confluence in 96-well plates and treated with a stress-inducing media of 2,2′-dithiodipyridine (DTDP) at a concentration of 200 μM for 10 min (37 °C, 95% air, 5% CO2). This was subsequently replaced with fresh media (110 μL) containing the Ez-Cytox reagent (WST-1, Daeil Lab, Korea) and cells were then incubated for 4 h under normal cell culture conditions. After this incubation, the absorbance of each well on the plate was measured at a wavelength of 450 nm using a VersaMax multiwall plate spectrophotometer (Molecular Devices, Sunnyvale, CA, USA).

### 4.10. Statistical Analysis

Data are expressed as mean ± S.E.M. for all three independent experiments, unless otherwise specified for each figure. The Student’s t test was used to determine significant difference between means and *p* < 0.05 was considered statistically significant.

## Abbreviations

K_v_2.1	Voltage-dependent potassium channel K_v_2.1
mAb	Monoclonal antibody
RBM	Rat brain membrane
DTDP	2,2′-dithiodipyridine
WST-1	Ez-Cytox reagent
SFK	Src kinase family kinases
